# Genetically determined telomere length as a risk factor for hematological malignancies: evidence from Mendelian randomization analysis

**DOI:** 10.18632/aging.205625

**Published:** 2024-03-06

**Authors:** Tian Fang, Zhihao Zhang, Kexing Ren, Liqun Zou

**Affiliations:** 1 Department of Medical Oncology, Cancer Center, West China Hospital, Sichuan University, Chengdu, China; 2 Department of Breast Center, West China Hospital, Sichuan University, Chengdu, China

**Keywords:** Mendelian randomization, telomere length, hematopoietic malignancies, lymphoma, causal relationship

## Abstract

Background: Over the past years, the exact correlation between telomere length and hematological malignancies was still not fully understood.

Methods: We performed a two-sample Mendelian randomization study to investigate the causal relationship between telomere length and hematological malignancies. We selected genetic instruments associated with telomere length. The genetic associations for lymphoid and hematopoietic malignant neoplasms were obtained from the most recent publicly accessible FinnGen study R9 data. Inverse variant weighted (IVW) analysis was adopted as the primary method, and we also performed the weighted-median method and the MR-Egger, and MRPRESSO methods as sensitive analysis.

Results: Significant associations have been observed between telomere length and primary lymphoid (IVW: OR = 1.52, *P* = 2.11 × 10^−6^), Hodgkin lymphoma (IVW: OR = 1.64, *P* = 0.014), non-Hodgkin lymphoma (IVW: OR = 1.70, *P* = 0.002), B-cell lymphoma (IVW: OR = 1.57, *P* = 0.015), non-follicular lymphoma (IVW: OR = 1.58, *P* = 1.7 × 10^−3^), mantle cell lymphoma (IVW: OR = 3.13, *P* = 0.003), lymphoid leukemia (IVW: OR = 2.56, *P* = 5.92E-09), acute lymphocytic leukemia (IVW: OR = 2.65, *P* = 0.021) and chronic lymphocytic leukemia (IVW: OR = 2.80, *P* = 8.21 × 10^−6^), along with multiple myeloma (IVW: OR = 1.85, *P* = 0.016).

Conclusion: This MR study found a significant association between telomere length and a wide range of hematopoietic malignancies. But no substantial impact of lymphoma and hematopoietic malignancies on telomere length has been detected.

## INTRODUCTION

Malignant tumors of the hematopoietic system occur in the blood-forming tissues. In 2020, more than 1.2 million people worldwide were diagnosed with malignant tumors of the hematopoietic system, and over 700,000 people died from these malignancies [1]. Hematological malignancies are broadly categorized into lymphoma, which originates from the lymphatic system; multiple myeloma (MM), which affects plasma cells in the bone marrow; and leukemia, which impacts cells in the bone marrow or blood [2]. Currently, many observational studies have investigated the etiology of hematological malignancies, including factors such as air pollution, chlamydia contamination, and dietary habits, among others [3, 4]. However, due to the multitude of confounding factors in retrospective studies, many causative factors still cannot be conclusively determined.

Telomeres are natural ends of chromosomes characterized by variable numbers of TTAGGG repeat sequences and associated proteins [5]. The role of telomeres in human health and disease is still not fully understood. But more and more studies have demonstrated that telomeres play a crucial role in the development and progression of cancer [6]. Several studies have found that longer telomeres may be associated with an increased risk of various cancers, including melanoma, acute myeloid leukemia, and chronic lymphocytic leukemia in cancer-prone families [7–9]. Bao et al. found that longer telomeres in leukocytes were identified as important risk factors for the development of myeloproliferative neoplasms [10]. However, several studies found that starting life with shorter telomeres may increases the risk of cancer [11]. The possible reason for generating these opposing views is the insufficient research methods.

Mendelian randomization (MR) uses genetic variation as an instrumental variable (IV), which has advantages compared with other research methods [12]. This approach utilizes genetic variants associated with the exposure of interest to estimate the causal effect on the outcome, thereby providing valuable insights into disease etiology [13–15]. In recent years, there has been growing interest in exploring the potential association between telomere length and other type of carcinomas using Mendelian randomization analysis [16]. However, the precise causal relationship between telomere length and the hematopoietic malignancies remains unclear.

This Mendelian randomization study aims to investigate the causal relationship between telomere length and hematopoietic malignancies. By utilizing large-scale genome-wide association studies (GWAS) data and applying rigorous statistical methods, we seek to provide robust evidence regarding the role of telomeres in hematopoietic malignancies and may have implications for risk prediction, prevention, and potentially targeted therapies.

## MATERIALS AND METHODS

### Study design

**Figure 1:**
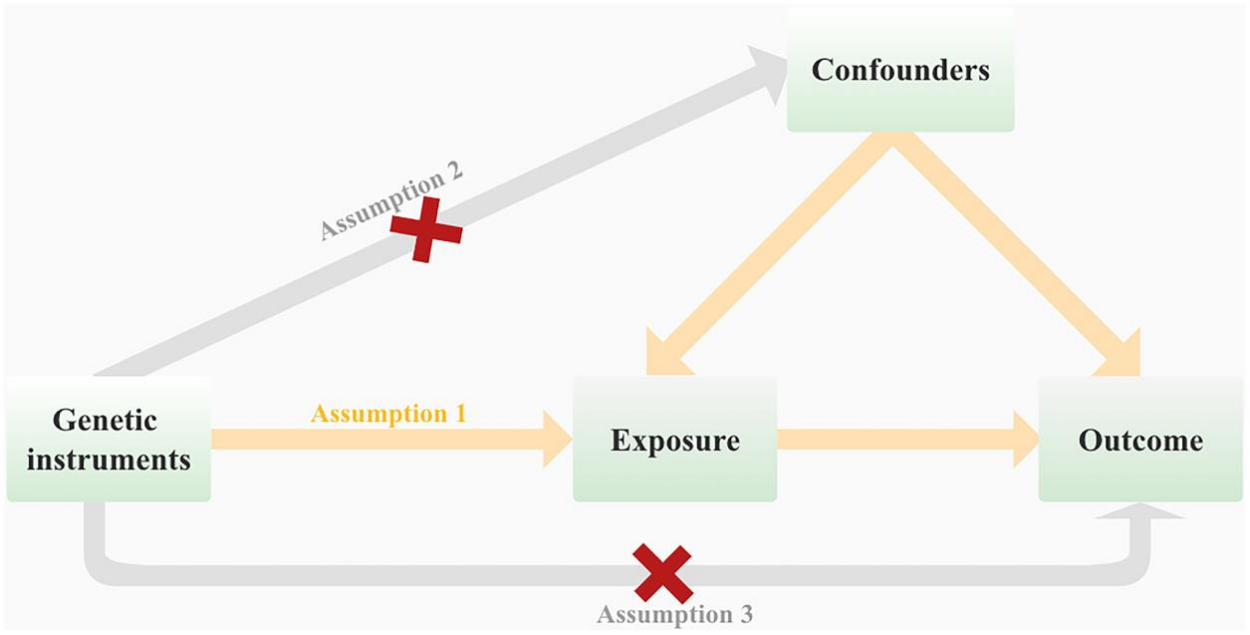
Mendelian randomization (MR) analysis is based on three fundamental assumptions at its core.

**Figure 2:**
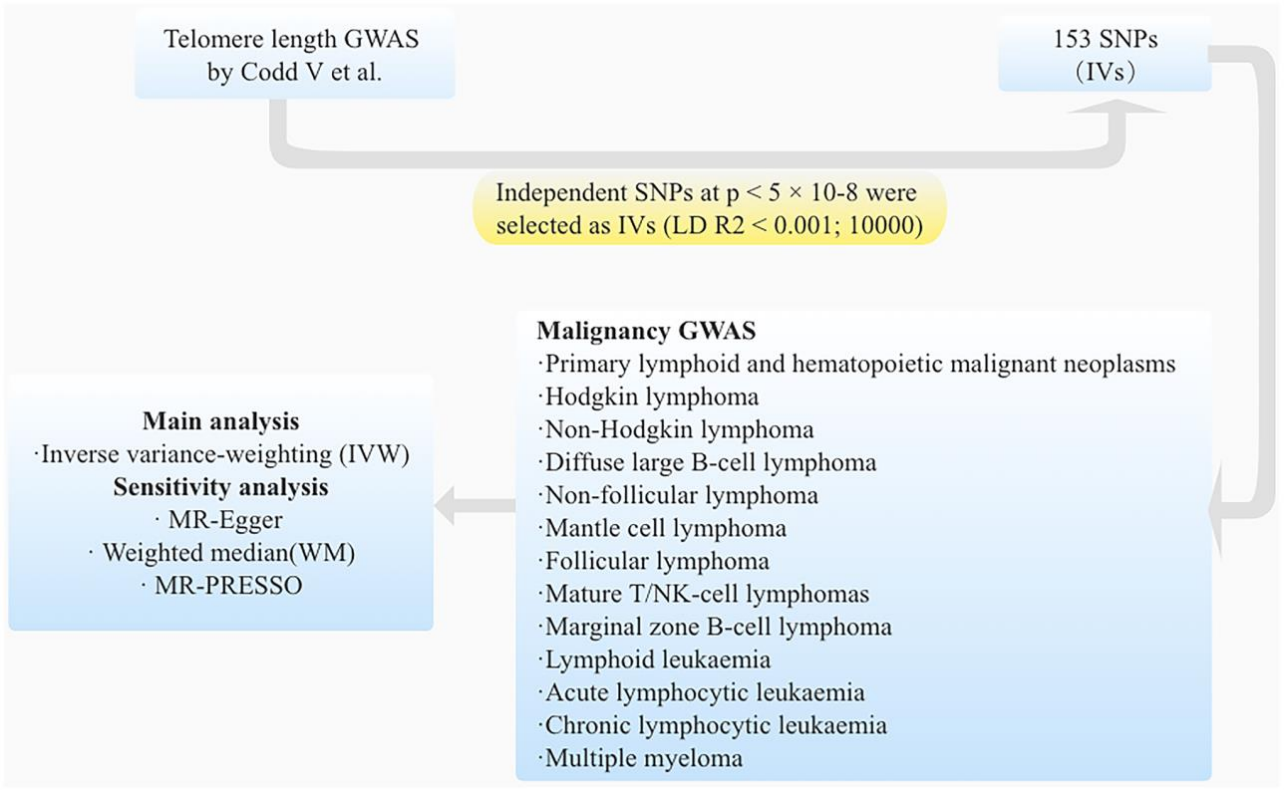
The flow diagram illustrates the sequential process of the MR study.

We performed a two-sample Mendelian randomization (MR) study to investigate the causal association between telomere length and hematological malignancies. As shown in Figure 1, in order for genetic variation to serve as a valid instrumental variable, it must adhere to three fundamental principles: (1) Genetic variants exhibit a robust correlation with the exposure of interest. (2) Genetic variants are not associated with potential confounders. (3) Genetic variants do not exert a direct influence on the outcome of interest. [17]. Figure 2 provides an overview of the study design. We reported this study according to the Strengthening the Reporting of Observational Studies in Epidemiology using Mendelian Randomization (STROBE-MR) [18].

### Data sources of exposure

Data on the association between genetics and telomere length were extracted from a GWAS conducted on a European cohort comprising 472,174 individuals (study ID “ieu-b-4879” and can be downloaded from the IEU GWAS database (https://gwas.mrcieu.ac.uk/datasets/) [19]. All participants fell within the age range of 40-69 years, with approximately equal representation of males (45.8%) and females (54.2%). The quantification of telomere length was performed using a well-established quantitative PCR method, and multiple quality control measures were implemented to account for potential influences of ethnicity, gender, age, and technical variables, as delineated in a previous investigation.

We included SNPs reaching GWAS (GWAS *p* < 5 × 10−8). Then, these SNPs were clumped based on the linkage disequilibrium (r 2 <0.001; kb = 10000) in the given genome region. Additionally, potential weak IVs (F-statistics <10) were excluded from the final analysis, as determined by calculating the F-statistics. Moreover, any palindromic SNPs with ambiguous minor allele frequencies (A/T or C/G) were discarded. Subsequently, we removed SNPs directly associated with hematological malignancies and confounding factors such as BMI [20] and tobacco smoking [21] through PhenoScanner datasets (http://www.phenoscanner.medschl.cam.ac.uk/).

### Data sources of outcome

To investigate genetic associations with lymphoid and hematopoietic malignant neoplasms, we employed summary-level data obtained from the most recent publicly accessible R9 data release by Kurki et al. [22]. The FinnGen study is a comprehensive nationwide genetic investigation conducted in conjunction with electronic health records, aiming to collect genetic data. This study was adjusted for potential confounding factors including sex, age, genetic components, and genotyping batch.

Genetic associations with 13 lymphoid and hematopoietic malignant neoplasms GWAS databases were available from the FinnGen website (https://www.finngen.fi/en). Included outcomes were classified into five major categories according to the pathological pattern: (1) Primary lymphoid and hematopoietic malignant neoplasms (7519 cases and 299,952 controls); (2) HL (2602 cases and 299,952 controls); (3) NHL (1088 cases and 299,952 controls): FL (1081 cases and 299,952 controls), Non-follicular lymphoma (NFL) (2602 cases and 299,952 controls), DLBCL (1010 cases and 287,137 controls), Mature T/NK-cell lymphomas (335 cases and 299,952 controls), Mantle cell lymphoma (MCL) (119 cases and 287,173 controls), Marginal zone B-cell lymphoma (MZBL) (192 cases and 287,137 controls); and (4) Lymphoid leukaemia (1493 cases and 299,952 controls) (Acute lymphocytic leukaemia (ALL) (184 cases and 287,136 controls), Chronic lymphocytic leukaemia (CLL) (624 cases and 287,133 controls)); (5) Multiple myeloma (MM) (674 cases and 376,603 controls).

### Primary MR analysis

The Wald ratio was used to assess the effect of telomere length on lymphoid and hematopoietic malignant neoplasms for each SNP. All SNP effects were meta-analyzed by the inverse-variance weighted (IVW) method [14]. This study used the multiplicative random-effects IVW method as the main MR analysis. In order to examine the potential causal relationship between telomere length and lymphoid and hematopoietic malignant neoplasms, we conducted MR analyses employing four distinct methods: IVW, MR-Egger, MR-PRESSO, and weighted median. The IVW method assumes the absence of pleiotropy, wherein instrumental variables (IVs) solely affect telomere length and not through alternative pathways. The MR-Egger approach provides a valid estimate of causal effect [23]. For the weighted median approach to be applicable, it necessitates that at least half of the IVs are valid [24]. The MR-PRESSO method effectively identifies potential IV abnormalities and automatically eliminates them to ensure an unbiased causal effect estimation. To assess heterogeneity, Cochran’s *Q* test was performed. In cases where no heterogeneity was observed in the IVW analysis, the fixed-effect model was utilized; otherwise, the random-effect model was employed.

### Sensitivity analysis

**Figure 3:**
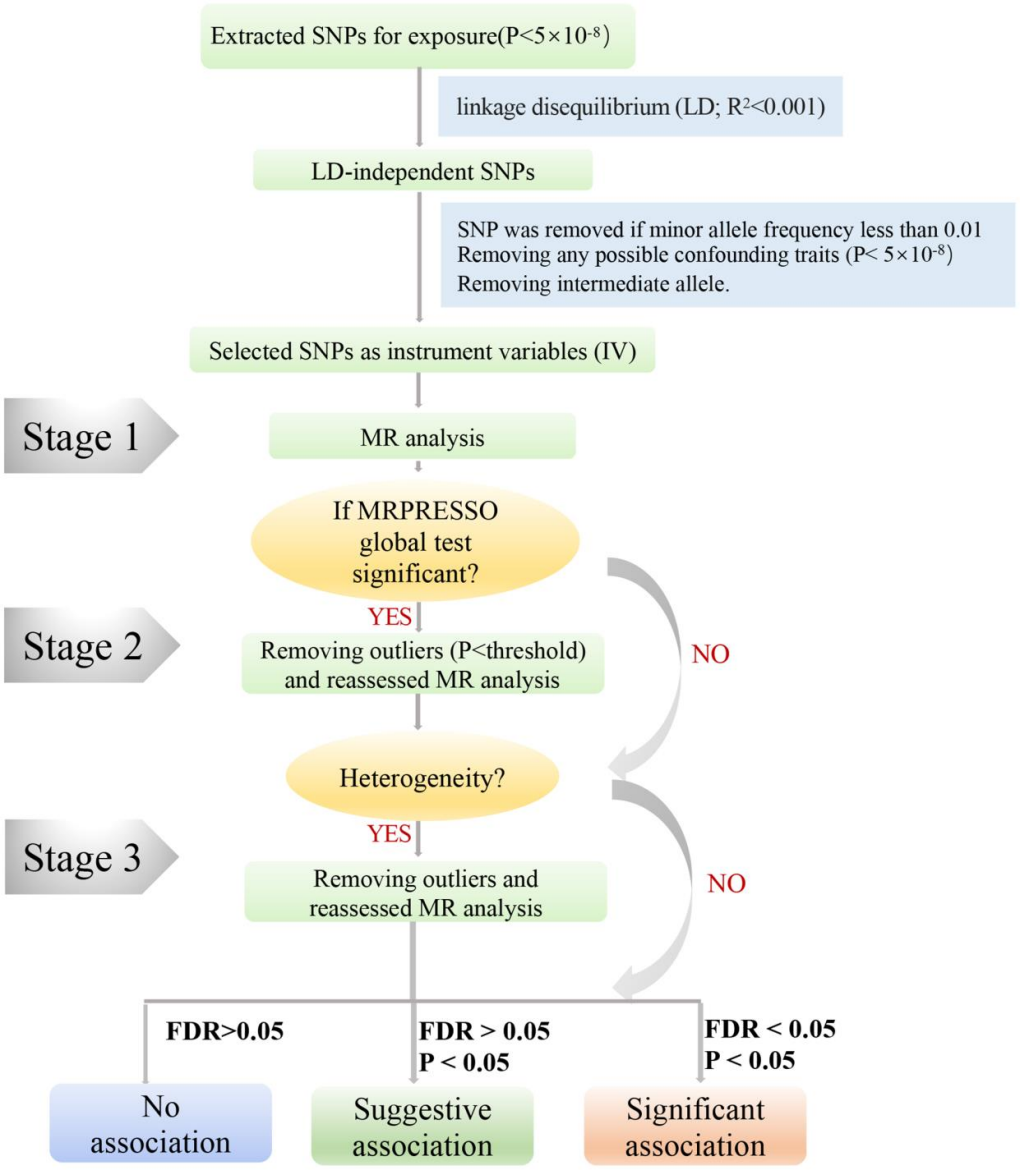
**A step-by-step flow chart demonstrates the analytical methods employed and outlines the sequential execution of MR analysis.** Step 1 involved conducting the MR analysis using the selected SNPs, followed by performing the MRPRESSO outlier test. If significance was detected (*P* < 0.05), we proceeded to step 2. In step 2, the MR analysis was reevaluated after removing all identified outliers (*P* < 0.05). Should heterogeneity persist, step 3 entailed excluding SNPs with a MR-PRESSO test *P*-value below 1 and reevaluating the MR analysis.

In sensitivity analyses, MR-Egger [23] and weighted median (WM) methods [24] were applied to account for horizontal pleiotropic effects. The MR-Egger method was based on the Instrument Strength Independent of Direct Effect (InSIDE) assumption, which often provides imprecise and low statistical power MR results, especially when meeting small sizes of SNPs (e.g., <10) [23]. In our MR study, MR-Egger was mainly used to detect pleiotropy; the value of the intercept term is far from zero, indicating horizontal pleiotropy (*P* < 0.05) [25]. The WM method was more reliable if more than 50% of SNPs were invalid instruments (e.g., due to pleiotropy) [26]. In addition, MR-PRESSO analysis was used to detect outliers, which can reduce heterogeneity by removing those outliers that may lead to heterogeneity (Figure 3) [27]. We performed leave-one-out method analysis to determine potentially influential SNPs by removing each SNP. We adjusted the multiple testing by false discovery rate (FDR).

### MR procedures

To ensure unbiased results by addressing potential heterogeneity, we followed a three-step approach in our study (Figure 3). In Step 1, we initially conducted MR analysis using the selected SNPs mentioned above, subsequently employing the MRPRESSO outlier test. If any outliers were detected (*P* < 0.05), we proceeded to Step 2. In Step 2, we reevaluated the MR analysis after excluding all outliers (*P* < 0.05). If heterogeneity persisted, we entered Step 3, wherein SNPs with a *P*-value less than 1 in the MR-PRESSO test were excluded, and the MR analysis was reevaluated. Furthermore, we exercised caution in interpreting the results if any potentially influential SNPs were identified through the leave-one-out test.

For our MR study, we utilized several R packages including “TwoSampleMR” [28], “MendelianRandomization” [24], and “MRPRESSO” [27] packages. The forestploter R packages were employed for data visualization. All statistical analyses were conducted using R software version 4.3.1 (R Foundation, Vienna, Austria, https://www.R-project.org/).

## RESULTS

We identified 34857 SNPs that showed a significant association with telomere length in the discovery cohort consisting of 472,174 European participants, as reported by Codd V et al. [19]. These SNPs reached the genome-wide significance level (*p*-value < 5 × 10−8). To ensure the independence of instrumental variables for telomere length (TL), SNPs in linkage disequilibrium (with r2 >0.001 and clump distance <10,000 kb) were excluded. Notably, rs7705526 was excluded due to its significant association with some hematological malignancies directly by screening PhenoScanner datasets (*P* < 5 × 10−8). Ultimately, 153 independent SNPs remained as instrumental variables. Supplementary Table 1 provides detailed information on the selected SNPs. The F statistics of these SNPs ranged from 29 to 1628, suggesting no weak instrumental variables exited [29]. The instrumental variables accounted for 3.36% of the variance in explaining the exposure.

### MR main analysis

**Figure 4:**
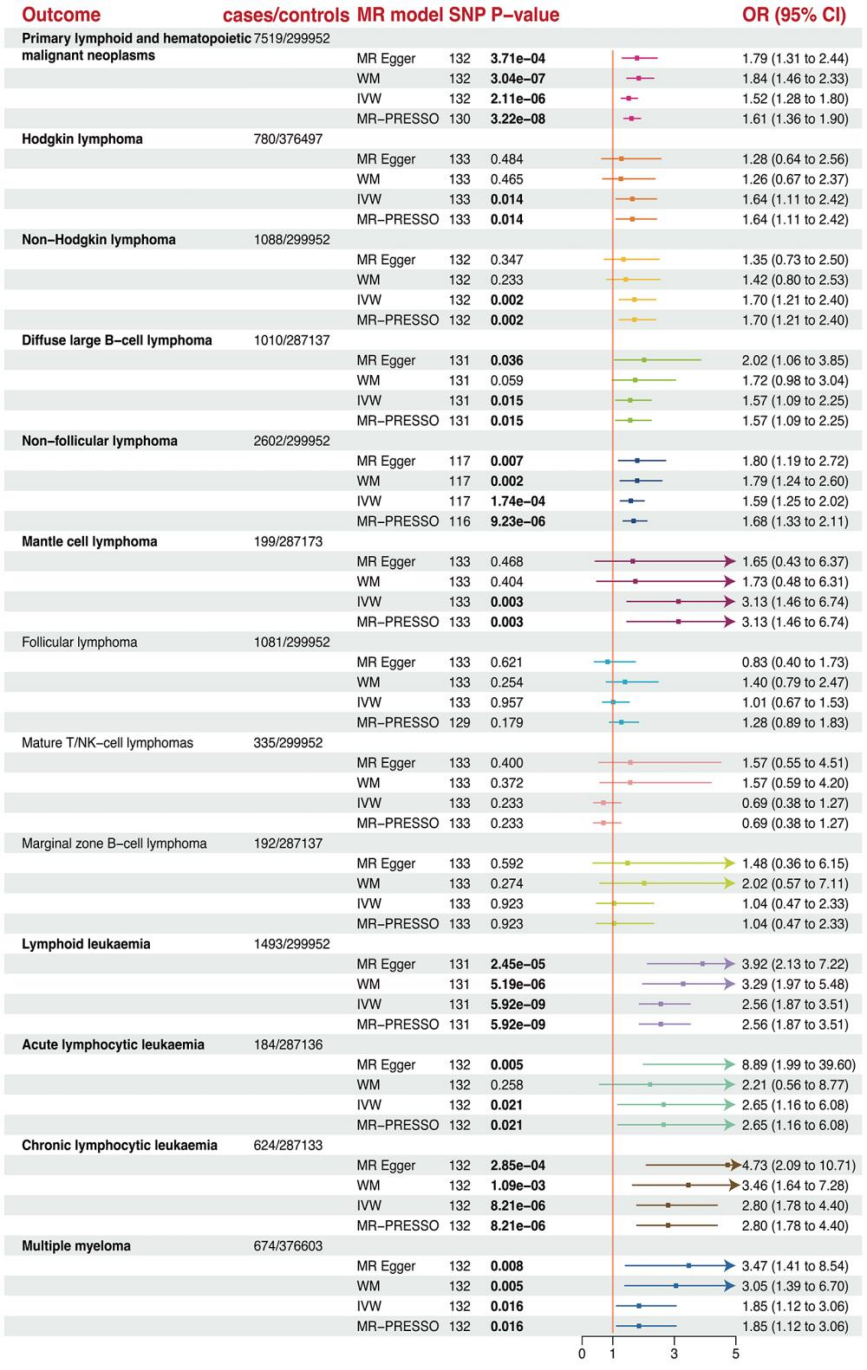
The forest plots revealed the causal association of telomere length with different lymphoma and hematopoietic malignancies.

Genetically predicted longer telomere length could increase the risk of all types of primary lymphoid, Hodgkin lymphoma (HL), non-Hodgkin lymphoma (NHL), diffuse large B-cell lymphoma (DLBCL), non-follicular lymphoma (NFL), mantle cell lymphoma (MCL), acute lymphoid leukemia (ALL), chronic lymphoid leukemia (CLL) after FDR control (FDR <0.05; Figure 4 and Table 1). Specifically, a 1-SD increase of telomere length could increase the risk of all types of primary lymphoid (OR = 1.52, *P* = 2.11×10^−6^) by 52%, HL (OR = 1.64, *P* = 0.014) by 64%, NHL (OR = 1.70, *P* = 0.002) by 70%, DLBCL (OR = 1.57, *P* = 0.015) by 57%, NFL (OR = 1.58, *P* = 1.73 × 10^−4^) by 58%, MCL (OR = 3.13, *P* = 0.003) by 213%, ALL (OR = 2.65, *P* = 0.021) by 165%, CLL (OR = 2.80, *P* = 8.21 × 10^−6^) by 180%, and Multiple myeloma 1.85 (OR = 1.85, *P* = 0.016) by 85%. However, telomere length was not associated with increase in odds of follicular lymphomas (FL), mature T/NK cell lymphoma, and marginal zone B-cell lymphoma (MZBL).

**Table 1:** False discovery rate adjusted *p*-values for the tested associations of telomere length and outcomes.

**Outcome**	**SNPs**	**Original *P*-value**	**Benjamini-Hochberg adjusted *P*-value**	**Significant using an FDR of 0.05?**
Primary lymphoid and hematopoietic malignant neoplasms	132	2.11E-06	1.37E-05	Yes
Hodgkin lymphoma	117	0.014	0.026	Yes
Non-Hodgkin lymphoma	132	0.002	0.006	Yes
Diffuse large B-cell lymphoma	133	0.015	0.019	Yes
Non-follicular lymphoma	131	0.000	0.001	Yes
Mantle cell lymphoma	133	0.003	0.007	Yes
Follicular lymphoma	133	0.957	0.957	No
Mature T/NK-cell lymphomas	133	0.233	0.276	No
Marginal zone B-cell lymphoma	133	0.923	1.000	No
Lymphoid leukaemia	131	5.92E-09	7.69E-08	Yes
Acute lymphocytic leukaemia	132	2.09E-02	3.02E-02	Yes
Chronic lymphocytic leukaemia	132	0.000	0.000	Yes
Multiple myeloma	132	0.016	0.026	Yes

### Sensitivity analysis

All WM and MR-Egger sensitivity analyses were directionally consistent in the IVW results except for FL and mature T/NK-cell lymphomas. The MR-PRESSO results suggested that the causal relationship still holds after removing outliers. No heterogeneity was detected except for primary lymphoid and FL. Furthermore, no horizontal pleiotropy was observed in this MR analysis across all subsets (Supplementary Table 2). Moreover, Leave-one-out analysis revealed that no SNP drove the results (Supplementary Figure 1).

To further investigate the causal relationship, we examined the effect of these malignancies as a risk factor for telomere length, thus ruling out the possibility of a bidirectional causal effect between telomere length and all types of lymphoma and lymphoid leukemia. Different SNPs associated with various lymphomas were considered, but no significant effect was found under any of the MR models. These results demonstrate the credibility of our conclusions.

## DISCUSSION

The objective of this study is to investigate the association between telomere length and various hematopoietic malignancies by MR analysis. Through our research, we found that telomere length may increase the risk of primary lymphoid, Hodgkin lymphoma, non-Hodgkin lymphoma, DLBCL, NFL, MCL, ALL, CLL and multiple myeloma. These findings were supported by rigorous statistical analysis, with a corrected FDR of less than 0.05. Furthermore, this study demonstrated no homogeneity and horizontal pleiotropy across all investigations.

Telomere length plays a critical role in maintaining genomic stability and preventing cellular senescence or apoptosis [30, 31]. Shortened telomeres are commonly observed in various cancer types and are generally associated with genomic instability and tumor progression [32–34]. Our findings add to the existing body of evidence by highlighting the importance of telomere length in hematopoietic malignancies. Previous studies have concluded that blood malignancies (NHL, MCL, ALL, CLL) have shorter telomere lengths than the control group [35–39]. Roos et al. found that shortened telomere length in chronic lymphocytic leukemia (CLL) patients is consistent with other classic biological factors of CLL, including unmutated immunoglobulin heavy chain variable region genes (UM-IGVH), positive CD38 and ZAP-70 (>30%), and short lymphocyte doubling time (<6 months) [40]. In a study conducted by Sellmann et al., a correlation was observed between the frequency of IGHV gene mutations and the length of telomeres [41]. The study conducted on CLL patients indicated that those with reduced telomere length demonstrated poorer clinical outcomes, including decreased progression-free survival (PFS) and overall survival (OS) [42]. However, their findings do not elucidate a causal relationship between telomere length and hematologic malignancies. Furthermore, it is worth noting that Furtado et al. [43] suggest that telomere shortening is an early event in the development of leukemia, as short telomeres are already present in small abnormal B-cell clones in monoclonal B-cell lymphocytosis. This disease precedes chronic lymphocytic leukemia, consistent with the causal relationship we deduced through Mendelian randomization. Several underlying mechanisms could explain the observed association between telomere length and hematopoietic cancers. One possibility is that telomere dysfunction is directly involved in the initiation and progression of these malignancies. Shortened telomeres may lead to chromosomal abnormalities, DNA damage, and genomic instability, ultimately contributing to the development of cancer cells. Additionally, alterations in telomerase activity or mutations in genes involved in telomere regulation could contribute to telomere length abnormalities. Telomerase, the enzyme responsible for adding telomeric repeats, is often upregulated in cancer cells, allowing them to maintain or even lengthen their telomeres [44]. Dysregulation of telomerase activity could lead to differences in telomere length among individuals affecting their susceptibility to hematopoietic malignancies [30, 31].

Currently, research on telomere therapy is still in the early stages, and there is no definitive treatment plan. However, some studies have begun to explore potential treatment methods. One possible treatment approach is to extend telomere length through stem cell transplantation. Researchers have found that during the process of differentiation, stem cells can restore telomere length, which may be helpful in treating certain telomere shortening-related diseases [45]. In addition, certain drugs and compounds are being studied for their potential in intervening with telomere length. For example, some anti-aging compounds are speculated to have potential telomere protection effects, such as telomerase activators, antioxidants, and certain vitamins [46–48]. However, it should be noted that the relationship between telomere length and various diseases is complex. Treating telomere length involves considering the cellular environment, genetic factors, and other relevant factors [16]. Telomere therapy is still in the research stage and requires further clinical trials and studies to validate its safety, efficacy, and applicability.

Importantly, our findings have clinical implications for the diagnosis, prognosis, and treatment of hematopoietic malignancies. Telomere length could serve as a potential biomarker for disease risk assessment, allowing for early detection and intervention. Moreover, telomere length could be used to predict treatment response and patient outcomes, enabling personalized therapeutic strategies [49].

While this study provides valuable insights, there are certain limitations that should be acknowledged. Genetic and environmental factors that influence telomere length were not extensively investigated in this study. Further research is needed to elucidate these factors and their interactions.

However, there are some concomitant limitations in our study. First, due to the unavailability of individual-level data, we can only perform causal association MR analysis and cannot further examine the sensitivity and specificity of the outcomes. Additionally, the FinnGen database does not disclose detailed disease diagnostic information, which may introduce errors in our phenotypic analysis. However, the FinnGen database links genotypes with specific data using unique national identification numbers, and the disease classification is primarily based on ICD. Therefore, the possibility of misclassification influencing the outcomes is likely to be small. Second, further investigation into the direct impact of telomerase activity on hematological tumor development is necessary as telomere length is primarily influenced by telomerase. This research may provide new insights into the mechanisms through which telomeres contribute to cancer development. However, due to the lack of comprehensive telomerase-related GWAS (genome-wide association studies) at present, we are currently unable to analyze the relationship between telomerase and hematological tumors. In the future, relevant studies will be necessary. Third, a significant portion of the participants included in this investigation were of European origin; hence, it is not possible to extrapolate our results to encompass all racial groups.

## CONCLUSION

In conclusion, our study found that telomere length is a risk factor for a wide ride of hematopoietic malignancies. Understanding the role of telomere length in the pathogenesis of these cancers could pave the way for innovative diagnostic and therapeutic approaches. Further investigation into the underlying mechanisms and the identification of specific biomarkers associated with telomere length may contribute to improved clinical management and patient outcomes in hematopoietic malignancies.

## Supplementary Materials

Supplementary Figure 1

Supplementary Table 1

Supplementary Table 2

## References

[1] Andrades A, Peinado P, Alvarez-Perez JC, Sanjuan-Hidalgo J, García DJ, Arenas AM, Matia-González AM, Medina PP. SWI/SNF complexes in hematological malignancies: biological implications and therapeutic opportunities. Mol Cancer. 2023; 22:39. 10.1186/s12943-023-01736-8 36810086 PMC9942420

[2] Smith A, Howell D, Crouch S, Painter D, Blase J, Wang HI, Hewison A, Bagguley T, Appleton S, Kinsey S, Burton C, Patmore R, Roman E. Cohort Profile: The Haematological Malignancy Research Network (HMRN): a UK population-based patient cohort. Int J Epidemiol. 2018; 47:700. 10.1093/ije/dyy044 29618056 PMC6005016

[3] Drexler HG, Dirks WG, MacLeod RA, Uphoff CC. False and mycoplasma-contaminated leukemia-lymphoma cell lines: time for a reappraisal. Int J Cancer. 2017; 140:1209–14. 10.1002/ijc.30530 27870004

[4] Taj T, Chen J, Rodopoulou S, Strak M, de Hoogh K, Poulsen AH, Andersen ZJ, Bellander T, Brandt J, Zitt E, Fecht D, Forastiere F, Gulliver J, et al. Long-term exposure to ambient air pollution and risk of leukemia and lymphoma in a pooled European cohort. Environ Pollut. 2024; 343:123097. 10.1016/j.envpol.2023.123097 38065336

[5] de Lange T. Shelterin-Mediated Telomere Protection. Annu Rev Genet. 2018; 52:223–47. 10.1146/annurev-genet-032918-021921 30208292

[6] Lansdorp PM. Telomeres, aging, and cancer: the big picture. Blood. 2022; 139:813–21. 10.1182/blood.2021014299 35142846 PMC8832478

[7] McNally EJ, Luncsford PJ, Armanios M. Long telomeres and cancer risk: the price of cellular immortality. J Clin Invest. 2019; 129:3474–81. 10.1172/JCI120851 31380804 PMC6715353

[8] Haycock PC, Burgess S, Nounu A, Zheng J, Okoli GN, Bowden J, Wade KH, Timpson NJ, Evans DM, Willeit P, Aviv A, Gaunt TR, Hemani G, et al, and Telomeres Mendelian Randomization Collaboration. Association Between Telomere Length and Risk of Cancer and Non-Neoplastic Diseases: A Mendelian Randomization Study. JAMA Oncol. 2017; 3:636–51. 10.1001/jamaoncol.2016.5945 28241208 PMC5638008

[9] Schmutz I, Mensenkamp AR, Takai KK, Haadsma M, Spruijt L, de Voer RM, Choo SS, Lorbeer FK, van Grinsven EJ, Hockemeyer D, Jongmans MC, de Lange T. *TINF2* is a haploinsufficient tumor suppressor that limits telomere length. Elife. 2020; 9:e61235. 10.7554/eLife.61235 33258446 PMC7707837

[10] Bao EL, Nandakumar SK, Liao X, Bick AG, Karjalainen J, Tabaka M, Gan OI, Havulinna AS, Kiiskinen TTJ, Lareau CA, de Lapuente Portilla AL, Li B, Emdin C, et al, and FinnGen, and 23andMe Research Team. Inherited myeloproliferative neoplasm risk affects haematopoietic stem cells. Nature. 2020; 586:769–75. 10.1038/s41586-020-2786-7 33057200 PMC7606745

[11] Nassour J, Schmidt TT, Karlseder J. Telomeres and Cancer: Resolving the Paradox. Annu Rev Cancer Biol. 2021; 5:59–77. 10.1146/annurev-cancerbio-050420-023410 34532611 PMC8442540

[12] Boyko EJ. Observational research--opportunities and limitations. J Diabetes Complications. 2013; 27:642–8. 10.1016/j.jdiacomp.2013.07.007 24055326 PMC3818421

[13] Martín-Masot R, Herrador-López M, Navas-López VM, Carmona FD, Nestares T, Bossini-Castillo L. Celiac Disease Is a Risk Factor for Mature T and NK Cell Lymphoma: A Mendelian Randomization Study. Int J Mol Sci. 2023; 24:7216. 10.3390/ijms24087216 37108375 PMC10139431

[14] Burgess S, Butterworth A, Thompson SG. Mendelian randomization analysis with multiple genetic variants using summarized data. Genet Epidemiol. 2013; 37:658–65. 10.1002/gepi.21758 24114802 PMC4377079

[15] Smith GD, Ebrahim S. 'Mendelian randomization': can genetic epidemiology contribute to understanding environmental determinants of disease? Int J Epidemiol. 2003; 32:1–22. 10.1093/ije/dyg070 12689998

[16] Ye Q, Apsley AT, Etzel L, Hastings WJ, Kozlosky JT, Walker C, Wolf SE, Shalev I. Telomere length and chronological age across the human lifespan: A systematic review and meta-analysis of 414 study samples including 743,019 individuals. Ageing Res Rev. 2023; 90:102031. 10.1016/j.arr.2023.102031 37567392 PMC10529491

[17] Davey Smith G, Hemani G. Mendelian randomization: genetic anchors for causal inference in epidemiological studies. Hum Mol Genet. 2014; 23:R89–98. 10.1093/hmg/ddu328 25064373 PMC4170722

[18] Skrivankova VW, Richmond RC, Woolf BAR, Davies NM, Swanson SA, VanderWeele TJ, Timpson NJ, Higgins JPT, Dimou N, Langenberg C, Loder EW, Golub RM, Egger M, et al. Strengthening the reporting of observational studies in epidemiology using mendelian randomisation (STROBE-MR): explanation and elaboration. BMJ. 2021; 375:n2233. 10.1136/bmj.n2233 34702754 PMC8546498

[19] Codd V, Wang Q, Allara E, Musicha C, Kaptoge S, Stoma S, Jiang T, Hamby SE, Braund PS, Bountziouka V, Budgeon CA, Denniff M, Swinfield C, et al. Polygenic basis and biomedical consequences of telomere length variation. Nat Genet. 2021; 53:1425–33. 10.1038/s41588-021-00944-6 34611362 PMC8492471

[20] Strongman H, Brown A, Smeeth L, Bhaskaran K. Body mass index and Hodgkin's lymphoma: UK population-based cohort study of 5.8 million individuals. Br J Cancer. 2019; 120:768–70. 10.1038/s41416-019-0401-1 30808991 PMC6461799

[21] Sergentanis TN, Kanavidis P, Michelakos T, Petridou ET. Cigarette smoking and risk of lymphoma in adults: a comprehensive meta-analysis on Hodgkin and non-Hodgkin disease. Eur J Cancer Prev. 2013; 22:131–50. 10.1097/CEJ.0b013e328355ed08 22759975

[22] Kurki MI, Karjalainen J, Palta P, Sipilä TP, Kristiansson K, Donner KM, Reeve MP, Laivuori H, Aavikko M, Kaunisto MA, Loukola A, Lahtela E, Mattsson H, et al. FinnGen provides genetic insights from a well-phenotyped isolated population. Nature. 2023; 613:508–18. 10.1038/s41586-022-05473-8 36653562 PMC9849126

[23] Burgess S, Thompson SG. Interpreting findings from Mendelian randomization using the MR-Egger method. Eur J Epidemiol. 2017; 32:377–89. 10.1007/s10654-017-0255-x 28527048 PMC5506233

[24] Yavorska OO, Burgess S. MendelianRandomization: an R package for performing Mendelian randomization analyses using summarized data. Int J Epidemiol. 2017; 46:1734–9. 10.1093/ije/dyx034 28398548 PMC5510723

[25] Bowden J, Davey Smith G, Burgess S. Mendelian randomization with invalid instruments: effect estimation and bias detection through Egger regression. Int J Epidemiol. 2015; 44:512–25. 10.1093/ije/dyv080 26050253 PMC4469799

[26] Bowden J, Davey Smith G, Haycock PC, Burgess S. Consistent Estimation in Mendelian Randomization with Some Invalid Instruments Using a Weighted Median Estimator. Genet Epidemiol. 2016; 40:304–14. 10.1002/gepi.21965 27061298 PMC4849733

[27] Verbanck M, Chen CY, Neale B, Do R. Detection of widespread horizontal pleiotropy in causal relationships inferred from Mendelian randomization between complex traits and diseases. Nat Genet. 2018; 50:693–8. 10.1038/s41588-018-0099-7 29686387 PMC6083837

[28] Hemani G, Zheng J, Elsworth B, Wade KH, Haberland V, Baird D, Laurin C, Burgess S, Bowden J, Langdon R, Tan VY, Yarmolinsky J, Shihab HA, et al. The MR-Base platform supports systematic causal inference across the human phenome. Elife. 2018; 7:e34408. 10.7554/eLife.34408 29846171 PMC5976434

[29] Pierce BL, Ahsan H, Vanderweele TJ. Power and instrument strength requirements for Mendelian randomization studies using multiple genetic variants. Int J Epidemiol. 2011; 40:740–52. 10.1093/ije/dyq151 20813862 PMC3147064

[30] Shay JW, Wright WE. Telomeres and telomerase: three decades of progress. Nat Rev Genet. 2019; 20:299–309. 10.1038/s41576-019-0099-1 30760854

[31] Vakonaki E, Tsiminikaki K, Plaitis S, Fragkiadaki P, Tsoukalas D, Katsikantami I, Vaki G, Tzatzarakis MN, Spandidos DA, Tsatsakis AM. Common mental disorders and association with telomere length. Biomed Rep. 2018; 8:111–6. 10.3892/br.2018.1040 29435268 PMC5778888

[32] Tsatsakis A, Oikonomopoulou T, Nikolouzakis TK, Vakonaki E, Tzatzarakis M, Flamourakis M, Renieri E, Fragkiadaki P, Iliaki E, Bachlitzanaki M, Karzi V, Katsikantami I, Kakridonis F, et al. Role of telomere length in human carcinogenesis (Review). Int J Oncol. 2023; 63:78. 10.3892/ijo.2023.5526 37232367 PMC10552730

[33] Bernal A, Tusell L. Telomeres: Implications for Cancer Development. Int J Mol Sci. 2018; 19:294. 10.3390/ijms19010294 29351238 PMC5796239

[34] Schratz KE, Gaysinskaya V, Cosner ZL, DeBoy EA, Xiang Z, Kasch-Semenza L, Florea L, Shah PD, Armanios M. Somatic reversion impacts myelodysplastic syndromes and acute myeloid leukemia evolution in the short telomere disorders. J Clin Invest. 2021; 131:e147598. 10.1172/JCI147598 34343137 PMC8439599

[35] Haydeé Cottliar AS, Noriega MF, Narbaitz M, Rodríguez A, Slavutsky IR. Association between telomere length and BCL2 gene rearrangements in low- and high-grade non-Hodgkin lymphomas. Cancer Genet Cytogenet. 2006; 171:1–8. 10.1016/j.cancergencyto.2006.05.016 17074584

[36] Widmann TA, Herrmann M, Taha N, König J, Pfreundschuh M. Short telomeres in aggressive non-Hodgkin's lymphoma as a risk factor in lymphomagenesis. Exp Hematol. 2007; 35:939–46. 10.1016/j.exphem.2007.03.009 17533048

[37] Adamson DJ, King DJ, Haites NE. Significant telomere shortening in childhood leukemia. Cancer Genet Cytogenet. 1992; 61:204–6. 10.1016/0165-4608(92)90088-p 1638505

[38] Song DY, Kim JA, Jeong D, Yun J, Kim SM, Lim K, Park SN, Im K, Choi S, Yoon SS, Lee DS. Telomere length and its correlation with gene mutations in chronic lymphocytic leukemia in a Korean population. PLoS One. 2019; 14:e0220177. 10.1371/journal.pone.0220177 31335885 PMC6650075

[39] Jebaraj BM, Kienle D, Lechel A, Mertens D, Heuberger M, Ott G, Rosenwald A, Barth TF, Möller P, Zenz T, Döhner H, Stilgenbauer S. Telomere length in mantle cell lymphoma. Blood. 2013; 121:1184–7. 10.1182/blood-2012-08-452649 23243283

[40] Roos G, Kröber A, Grabowski P, Kienle D, Bühler A, Döhner H, Rosenquist R, Stilgenbauer S. Short telomeres are associated with genetic complexity, high-risk genomic aberrations, and short survival in chronic lymphocytic leukemia. Blood. 2008; 111:2246–52. 10.1182/blood-2007-05-092759 18045969

[41] Sellmann L, de Beer D, Bartels M, Opalka B, Nückel H, Dührsen U, Dürig J, Seifert M, Siemer D, Küppers R, Baerlocher GM, Röth A. Telomeres and prognosis in patients with chronic lymphocytic leukaemia. Int J Hematol. 2011; 93:74–82. 10.1007/s12185-010-0750-2 21203871

[42] Counter CM, Gupta J, Harley CB, Leber B, Bacchetti S. Telomerase activity in normal leukocytes and in hematologic malignancies. Blood. 1995; 85:2315–20. 10.1182/blood.V85.9.2315.bloodjournal8592315 7727765

[43] Furtado FM, Scheucher PS, Santana BA, Scatena NF, Calado RT, Rego EM, Matos DM, Falcão RP. Telomere length analysis in monoclonal B-cell lymphocytosis and chronic lymphocytic leukemia Binet A. Braz J Med Biol Res. 2017; 50:e6019. 10.1590/1414-431X20176019 28423121 PMC5441285

[44] Maciejowski J, de Lange T. Telomeres in cancer: tumour suppression and genome instability. Nat Rev Mol Cell Biol. 2017; 18:175–86. 10.1038/nrm.2016.171 28096526 PMC5589191

[45] Wang L, Xiao H, Zhang X, Wang C, Huang H. The role of telomeres and telomerase in hematologic malignancies and hematopoietic stem cell transplantation. J Hematol Oncol. 2014; 7:61. 10.1186/s13045-014-0061-9 25139287 PMC4237881

[46] Küçüksolak M, Yılmaz S, Ballar-Kırmızıbayrak P, Bedir E. Potent telomerase activators from a novel sapogenin via biotransformation utilizing Camarosporium laburnicola, an endophytic fungus. Microb Cell Fact. 2023; 22:66. 10.1186/s12934-023-02069-3 37024895 PMC10080871

[47] Duman S, Ekiz G, Yılmaz S, Yusufoglu H, Ballar Kırmızıbayrak P, Bedir E. Telomerase activators from 20(27)-octanor-cycloastragenol via biotransformation by the fungal endophytes. Bioorg Chem. 2021; 109:104708. 10.1016/j.bioorg.2021.104708 33621779

[48] Fragkiadaki P, Renieri E, Kalliantasi K, Kouvidi E, Apalaki E, Vakonaki E, Mamoulakis C, Spandidos DA, Tsatsakis A. Τelomerase inhibitors and activators in aging and cancer: A systematic review. Mol Med Rep. 2022; 25:158. 10.3892/mmr.2022.12674 35266017 PMC8941523

[49] Jones CH, Pepper C, Baird DM. Telomere dysfunction and its role in haematological cancer. Br J Haematol. 2012; 156:573–87. 10.1111/j.1365-2141.2011.09022.x 22233151

