# Supplementary material for: Genetically determined telomere length as a risk factor for hematological malignancies: evidence from Mendelian randomization analysis
Source: Aging (Albany NY). 2024 Mar 6;16(5):4684–98. doi: 10.18632/aging.205625 (PMC10968690; doi:10.18632/aging.205625)
Supplement: Supplementary Figure 1 [file aging-16-205625-s001.pdf]

SUPPLEMENTARY FIGURE

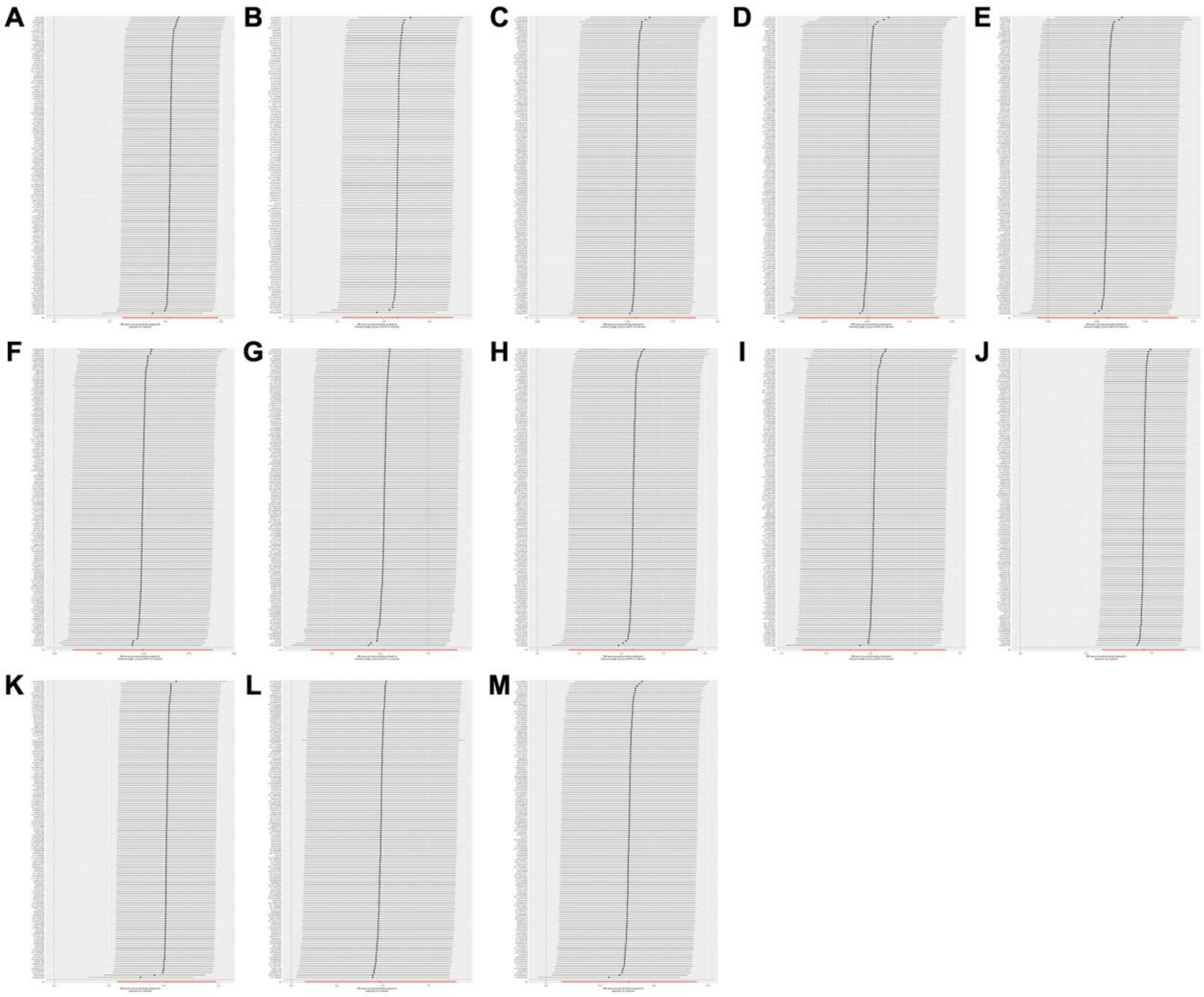

Supplementary Figure 1. Sensitivity analysis of the leave-one-out test for the MR analysis to assess the impact of telomere length on various cancer risks. (**A**) TL on primary lymphoid and hematopoietic malignant neoplasms. (**B**) TL on non-follicular lymphoma. (**C**) TL on non-Hodgkin lymphoma. (**D**) TL on follicular lymphoma. (**E**) TL on diffuse large B-cell lymphoma. (**F**) TL on Hodgkin lymphoma. (**G**) TL on mature T/NK-cell lymphomas. (**H**) TL on mantle cell lymphoma. (**I**) TL on marginal zone B-cell lymphoma. (**J**) TL on lymphoid leukaemia. (**K**) TL on chronic lymphocytic leukaemia. (**L**) TL on acute lymphocytic leukaemia. (**M**) TL on multiple myeloma.
