# Supplementary material for: Genetically determined telomere length as a risk factor for hematological malignancies: evidence from Mendelian randomization analysis
Source: Aging (Albany NY). 2024 Mar 6;16(5):4684–98. doi: 10.18632/aging.205625 (PMC10968690; doi:10.18632/aging.205625)
Supplement: Supplementary Table 1 [file aging-16-205625-s002.docx]

| Supplementary Table 1. The baseline characteristics of the selected SNPs in the IEU GWAS. | | | | | | | | | |  |
| --- | --- | --- | --- | --- | --- | --- | --- | --- | --- | --- |
| **SNP** | **Gene** | **Chr** | **Position** | **β** | **SE** | **P-value** | **EA** | **OA** | **EAF** | **F-statistics** |
| rs145114957 | RP4-561L24.3 | 1 | 94322469 | 0.027 | 0.005 | 4.60E-08 | G | C | 0.043 | 29.9 |
| rs2977608 | LINC01128 | 1 | 768253 | 0.013 | 0.002 | 3.00E-08 | C | A | 0.744 | 30.7 |
| rs3767952 | NFYC | 1 | 41231032 | 0.013 | 0.002 | 1.80E-08 | A | G | 0.227 | 31.7 |
| rs6659669 | GS1-279B7.1 | 1 | 185315067 | -0.012 | 0.002 | 1.10E-08 | T | C | 0.605 | 32.6 |
| rs41269079 | BEST4 | 1 | 45252015 | 0.015 | 0.003 | 1.70E-09 | A | T | 0.189 | 36.3 |
| rs139795227 | RPAP2 | 1 | 92842367 | 0.060 | 0.009 | 6.70E-12 | C | A | 0.014 | 47.1 |
| rs6587577 | POGZ | 1 | 151402045 | -0.018 | 0.003 | 4.80E-12 | G | A | 0.826 | 47.8 |
| rs11579626 | CHD1L | 1 | 146741960 | 0.027 | 0.004 | 1.30E-13 | C | A | 0.085 | 54.9 |
| rs4498805 | SLC16A4 | 1 | 110910397 | 0.015 | 0.002 | 5.70E-14 | T | G | 0.547 | 56.5 |
| rs66731853 | CDA | 1 | 20916238 | -0.018 | 0.002 | 1.50E-16 | A | G | 0.317 | 68.1 |
| rs6669563 | SPOCD1 | 1 | 32279629 | 0.018 | 0.002 | 2.10E-19 | A | G | 0.438 | 81.1 |
| rs11584821 | BCL2L15 | 1 | 114419489 | -0.031 | 0.003 | 3.00E-31 | T | C | 0.176 | 135.2 |
| rs932002 | PARP1 | 1 | 226577306 | -0.040 | 0.003 | 7.30E-47 | T | C | 0.151 | 206.7 |
| rs6751209 | THADA | 2 | 43588302 | -0.014 | 0.002 | 1.60E-08 | C | T | 0.204 | 32.0 |
| rs77732866 | LINC01122 | 2 | 58979879 | 0.018 | 0.003 | 9.20E-10 | A | G | 0.138 | 37.5 |
| rs376641875 | C2orf73 | 2 | 54622978 | -0.027 | 0.004 | 3.30E-10 | C | CATAA | 0.929 | 39.5 |
| rs2555104 | SMC6 | 2 | 17841243 | -0.014 | 0.002 | 6.60E-12 | C | A | 0.434 | 47.1 |
| rs56178008 | TRMT61B | 2 | 29098543 | 0.014 | 0.002 | 9.70E-13 | A | T | 0.437 | 50.9 |
| rs188918174 | ACYP2 | 2 | 54473646 | 0.040 | 0.005 | 1.20E-13 | T | C | 0.036 | 55.0 |
| rs17803849 | UNC80 | 2 | 210673445 | 0.027 | 0.002 | 4.20E-41 | T | C | 0.405 | 180.3 |
| rs202034370 | ACYP2 | 2 | 54488018 | 0.103 | 0.007 | 2.60E-56 | T | TA | 0.975 | 250.0 |
| rs11426156 | RPN1 | 3 | 128318179 | -0.012 | 0.002 | 2.20E-08 | T | TA | 0.400 | 31.3 |
| rs6790988 | SLC7A14 | 3 | 170263320 | 0.015 | 0.002 | 1.80E-10 | G | A | 0.742 | 40.7 |
| rs13062095 | RNU6-461P | 3 | 101267385 | 0.014 | 0.002 | 9.70E-11 | C | T | 0.328 | 41.9 |
| rs869785 | THRB | 3 | 24347800 | -0.015 | 0.002 | 4.40E-12 | C | T | 0.672 | 47.9 |
| rs112394943 | TUBB8P8 | 3 | 197842892 | -0.020 | 0.003 | 1.60E-12 | C | T | 0.163 | 49.9 |
| rs9878436 | CEP70 | 3 | 138244400 | -0.014 | 0.002 | 1.20E-12 | T | C | 0.434 | 50.5 |
| rs2230590 | MST1R | 3 | 49936102 | -0.016 | 0.002 | 3.60E-15 | C | T | 0.511 | 61.9 |
| rs6776756 | GATA2 | 3 | 128215821 | -0.017 | 0.002 | 1.10E-17 | A | G | 0.598 | 73.3 |
| rs4616688 | IFT80 | 3 | 160042459 | -0.017 | 0.002 | 4.50E-18 | T | G | 0.525 | 75.1 |
| rs78491606 | SHQ1 | 3 | 72891547 | -0.076 | 0.007 | 1.90E-24 | C | A | 0.018 | 104.1 |
| rs35446936 | ACTRT3 | 3 | 169486508 | -0.094 | 0.002 | 1.00E-200 | A | G | 0.244 | 1628.8 |
| rs10805346 | SLC2A9 | 4 | 9920347 | 0.012 | 0.002 | 7.00E-09 | C | T | 0.439 | 33.5 |
| rs35500378 | EXOSC9 | 4 | 122729413 | 0.014 | 0.002 | 2.00E-12 | CACTT | C | 0.611 | 49.5 |
| rs4695407 | OCIAD1 | 4 | 48843372 | 0.014 | 0.002 | 1.50E-12 | G | A | 0.508 | 50.1 |
| rs2282764 | MXD4 | 4 | 2255063 | -0.022 | 0.003 | 9.30E-15 | G | A | 0.142 | 60.0 |
| rs871134 | CCDC96 | 4 | 7044380 | -0.018 | 0.002 | 1.70E-19 | T | C | 0.569 | 81.6 |
| rs6536702 | NAF1 | 4 | 164028105 | 0.053 | 0.002 | 9.40E-111 | A | G | 0.775 | 500.0 |
| rs55747751 | HSPA4 | 5 | 132397351 | -0.021 | 0.004 | 1.70E-08 | A | G | 0.077 | 31.8 |
| rs73730598 | LINC02142 | 5 | 77973 | 0.027 | 0.004 | 4.70E-10 | A | G | 0.055 | 38.8 |
| rs141214782 | PAPD4 | 5 | 78954683 | -0.025 | 0.003 | 2.00E-13 | TTATC | T | 0.101 | 54.0 |
| rs28363070 | SLC6A3 | 5 | 1415068 | 0.076 | 0.010 | 3.50E-15 | A | G | 0.013 | 61.9 |
| rs6881568 | LINC02142 | 5 | 1670265 | 0.017 | 0.002 | 3.70E-16 | A | C | 0.363 | 66.4 |
| rs185174247 | UBE2D2 | 5 | 138914024 | 0.037 | 0.004 | 1.10E-17 | A | G | 0.056 | 73.4 |
| rs61748181 | TERT | 5 | 1294166 | -0.059 | 0.006 | 2.80E-23 | T | C | 0.029 | 98.8 |
| **SNP** | **Gene** | **Chr** | **Position** | **β** | **SE** | **P-value** | **EA** | **OA** | **EAF** | **F-statistics** |
| rs9398196 | CCDC162P | 6 | 109601554 | -0.014 | 0.002 | 9.50E-13 | G | A | 0.520 | 50.9 |
| rs142730696 | - | 6 | 26360443 | 0.022 | 0.003 | 6.30E-13 | TTTTTC | T | 0.864 | 51.7 |
| rs1611236 | - | 6 | 29748690 | -0.016 | 0.002 | 6.10E-14 | A | G | 0.327 | 56.3 |
| rs201558190 | - | 6 | 29877483 | -0.018 | 0.002 | 6.40E-17 | C | T | 0.363 | 69.8 |
| rs80324517 | RP3-416J7.5 | 6 | 204031 | 0.040 | 0.005 | 1.80E-17 | A | G | 0.048 | 72.3 |
| rs7772289 | ZFP57 | 6 | 28674322 | 0.018 | 0.002 | 1.70E-18 | T | G | 0.503 | 77.0 |
| rs2538745 | AC004980.1 | 7 | 76310784 | -0.013 | 0.002 | 3.10E-10 | C | T | 0.603 | 39.6 |
| rs11769630 | AC020743.3 | 7 | 50257703 | -0.026 | 0.004 | 4.30E-11 | A | T | 0.072 | 43.5 |
| rs117407747 | PIP5K1P2 | 7 | 159117178 | 0.045 | 0.006 | 1.80E-13 | T | C | 0.028 | 54.2 |
| rs13230646 | AC009508.1 | 7 | 23930316 | -0.017 | 0.002 | 8.90E-14 | C | T | 0.249 | 55.6 |
| rs117630647 | POT1-AS1 | 7 | 124779510 | 0.060 | 0.007 | 1.40E-16 | A | G | 0.021 | 68.4 |
| rs61405042 | AC093627.7 | 7 | 67200 | -0.050 | 0.006 | 8.50E-17 | T | C | 0.029 | 69.3 |
| rs2056726 | STAG3 | 7 | 99780283 | -0.023 | 0.002 | 7.90E-21 | A | G | 0.214 | 87.6 |
| rs4731541 | TNPO3 | 7 | 128678236 | -0.021 | 0.002 | 1.40E-23 | G | C | 0.625 | 100.2 |
| rs1985369 | PIP5K1P2 | 7 | 159119220 | -0.031 | 0.003 | 3.60E-25 | G | A | 0.868 | 107.4 |
| rs7790856 | POT1 | 7 | 124459852 | -0.044 | 0.002 | 1.80E-87 | T | C | 0.289 | 393.0 |
| rs1023767 | KIAA1429 | 8 | 95530969 | -0.018 | 0.002 | 5.00E-15 | A | G | 0.238 | 61.2 |
| rs11991877 | TMEM68 | 8 | 56664524 | -0.030 | 0.003 | 3.20E-21 | A | T | 0.889 | 89.4 |
| rs2306646 | XPO7 | 8 | 21846586 | -0.021 | 0.002 | 3.30E-25 | C | G | 0.559 | 107.6 |
| rs762679 | MCM4 | 8 | 48885436 | 0.031 | 0.003 | 1.40E-27 | A | T | 0.857 | 118.4 |
| rs10112752 | TERF1 | 8 | 73958718 | -0.029 | 0.002 | 9.50E-46 | A | G | 0.430 | 201.6 |
| rs4743037 | ZNF462 | 9 | 109639970 | 0.015 | 0.002 | 5.10E-10 | T | C | 0.231 | 38.6 |
| rs34896435 | - | 9 | 826585 | 0.015 | 0.002 | 6.40E-14 | G | C | 0.469 | 56.2 |
| rs11557154 | DCAF12 | 9 | 34107505 | -0.034 | 0.003 | 1.10E-30 | T | C | 0.130 | 132.6 |
| rs117034449 | RP11-302K17.4 | 10 | 103855348 | 0.037 | 0.007 | 2.10E-08 | A | G | 0.023 | 31.4 |
| rs6584579 | STN1 | 10 | 105645725 | 0.011 | 0.002 | 2.00E-08 | G | A | 0.399 | 31.5 |
| rs7099229 | NOC3L | 10 | 96134685 | -0.015 | 0.002 | 8.40E-12 | A | G | 0.273 | 46.7 |
| rs77231040 | RP11-127O4.3 | 10 | 106280527 | 0.099 | 0.013 | 2.00E-13 | C | G | 0.006 | 54.0 |
| rs10905255 | GDI2 | 10 | 5870267 | -0.018 | 0.002 | 2.60E-19 | T | G | 0.579 | 80.7 |
| rs12412214 | LINC01475 | 10 | 101276256 | -0.025 | 0.002 | 3.40E-28 | A | G | 0.280 | 121.2 |
| rs9419958 | STN1 | 10 | 105675946 | -0.081 | 0.003 | 2.60E-167 | C | T | 0.861 | 760.0 |
| rs6590343 | RP11-744N12.3 | 11 | 128500215 | 0.012 | 0.002 | 1.50E-09 | G | A | 0.516 | 36.5 |
| rs2293579 | PSMC3 | 11 | 47440758 | -0.013 | 0.002 | 3.30E-10 | A | G | 0.386 | 39.5 |
| rs10840270 | RPL23AP65 | 11 | 9629553 | 0.014 | 0.002 | 1.30E-11 | G | C | 0.656 | 45.8 |
| rs11212631 | C11orf65 | 11 | 108304509 | -0.019 | 0.003 | 4.70E-14 | C | T | 0.199 | 56.9 |
| rs939916 | BET1L | 11 | 202253 | 0.024 | 0.002 | 6.60E-29 | A | G | 0.670 | 124.5 |
| rs10768683 | HBB | 11 | 5247791 | 0.047 | 0.003 | 1.50E-64 | G | C | 0.841 | 287.8 |
| rs611646 | ATM | 11 | 108177097 | -0.037 | 0.002 | 3.50E-73 | A | T | 0.409 | 327.4 |
| rs17445108 | PTGES3 | 12 | 57082058 | -0.017 | 0.003 | 2.00E-08 | A | G | 0.127 | 31.5 |
| rs12369950 | KNOP1P1 | 12 | 24762109 | -0.018 | 0.003 | 8.00E-10 | C | T | 0.141 | 37.8 |
| rs1907702 | KITLG | 12 | 88955469 | 0.015 | 0.002 | 5.90E-10 | A | G | 0.767 | 38.3 |
| rs10845387 | LINC01252 | 12 | 11757743 | -0.014 | 0.002 | 1.50E-11 | A | G | 0.353 | 45.5 |
| rs10774624 | RP3-473L9.4 | 12 | 111833788 | 0.015 | 0.002 | 2.90E-13 | A | G | 0.533 | 53.2 |
| rs10773176 | ZCCHC8 | 12 | 122944713 | -0.017 | 0.002 | 5.20E-14 | G | A | 0.741 | 56.7 |
| rs79977579 | - | 12 | 54694560 | 0.028 | 0.003 | 2.30E-16 | A | C | 0.096 | 67.3 |
| rs28577594 | RILPL2 | 12 | 123895906 | 0.019 | 0.002 | 5.40E-17 | C | G | 0.710 | 70.2 |
| **SNP** | **Gene** | **Chr** | **Position** | **β** | **SE** | **P-value** | **EA** | **OA** | **EAF** | **F-statistics** |
| rs76666449 | SRSF9 | 12 | 120904895 | 0.030 | 0.003 | 8.20E-19 | C | T | 0.101 | 78.5 |
| rs670180 | RP11-475F14.1 | 13 | 71236611 | -0.012 | 0.002 | 1.20E-08 | A | T | 0.569 | 32.5 |
| rs9600019 | BORA | 13 | 73317585 | 0.013 | 0.002 | 2.40E-09 | T | C | 0.336 | 35.6 |
| rs1332941 | RN7SL597P | 13 | 41695100 | 0.026 | 0.003 | 5.90E-21 | G | A | 0.820 | 88.2 |
| rs113525195 | PSMB5 | 14 | 23499321 | -0.012 | 0.002 | 3.10E-08 | A | C | 0.290 | 30.6 |
| rs3093888 | PARP2 | 14 | 20812951 | -0.029 | 0.005 | 1.50E-10 | A | G | 0.051 | 41.0 |
| rs73581419 | RAB2B | 14 | 21941148 | 0.023 | 0.003 | 1.30E-12 | T | C | 0.107 | 50.3 |
| rs1957937 | TCL1A | 14 | 96181360 | 0.021 | 0.003 | 1.90E-14 | T | A | 0.160 | 58.7 |
| rs34550383 | - | 14 | 91971787 | -0.019 | 0.002 | 8.60E-22 | C | CT | 0.547 | 92.0 |
| rs45604339 | MAX | 14 | 65543102 | -0.020 | 0.002 | 4.30E-22 | T | C | 0.342 | 93.4 |
| rs137901416 | DCAF4 | 14 | 73418095 | 0.046 | 0.003 | 4.70E-43 | A | G | 0.100 | 189.2 |
| rs7164950 | MNS1 | 15 | 56775385 | 0.013 | 0.002 | 2.30E-10 | G | A | 0.406 | 40.2 |
| rs5742915 | PML | 15 | 74336633 | 0.019 | 0.002 | 1.60E-21 | C | T | 0.446 | 90.8 |
| rs17677991 | MGA | 15 | 42032383 | 0.022 | 0.002 | 4.40E-26 | G | C | 0.342 | 111.6 |
| rs11412296 | ATP8B4 | 15 | 50366116 | 0.033 | 0.002 | 1.40E-45 | T | TA | 0.759 | 200.8 |
| rs450962 | EIF3CL | 16 | 28413517 | 0.014 | 0.002 | 5.90E-09 | G | A | 0.284 | 33.9 |
| rs111950327 | LONP2 | 16 | 48283993 | 0.024 | 0.004 | 5.90E-09 | C | G | 0.064 | 33.9 |
| rs12932179 | RP11-77H9.8 | 16 | 9072085 | -0.014 | 0.002 | 1.80E-11 | G | A | 0.561 | 45.2 |
| rs12925933 | PRDM7 | 16 | 90141355 | -0.015 | 0.002 | 7.00E-12 | C | A | 0.662 | 47.0 |
| rs9940099 | - | 16 | 3613207 | -0.034 | 0.004 | 3.20E-16 | T | G | 0.063 | 66.7 |
| rs76219171 | PAPD5 | 16 | 50188929 | 0.036 | 0.004 | 7.80E-17 | A | G | 0.058 | 69.5 |
| rs182059586 | PARN | 16 | 14652220 | -0.057 | 0.007 | 4.90E-17 | C | T | 0.025 | 70.4 |
| rs80116508 | - | 16 | 3650970 | -0.035 | 0.004 | 2.00E-17 | A | G | 0.062 | 72.2 |
| rs56061761 | PDPR | 16 | 70187811 | -0.020 | 0.002 | 6.90E-20 | A | G | 0.333 | 83.4 |
| rs11117354 | BANP | 16 | 88092092 | 0.023 | 0.002 | 3.40E-26 | C | T | 0.697 | 112.1 |
| rs3785074 | TERF2 | 16 | 69406986 | 0.024 | 0.002 | 2.60E-27 | G | A | 0.290 | 117.2 |
| rs76065543 | RFWD3 | 16 | 74678063 | 0.034 | 0.003 | 4.20E-32 | T | C | 0.138 | 139.1 |
| rs2967355 | MPHOSPH6 | 16 | 82200103 | -0.046 | 0.002 | 4.00E-83 | C | A | 0.774 | 373.1 |
| rs12451892 | SGSM2 | 17 | 2247982 | -0.012 | 0.002 | 2.20E-08 | C | T | 0.381 | 31.3 |
| rs7221585 | AFMID | 17 | 76195153 | 0.014 | 0.002 | 6.70E-09 | T | C | 0.224 | 33.6 |
| rs7209057 | NOL11 | 17 | 65705530 | 0.012 | 0.002 | 5.70E-09 | A | G | 0.561 | 33.9 |
| rs111527438 | ADAP2 | 17 | 29252703 | 0.013 | 0.002 | 3.10E-09 | C | T | 0.351 | 35.1 |
| rs59409453 | SERPINF1 | 17 | 1666218 | 0.020 | 0.002 | 1.60E-18 | G | A | 0.731 | 77.1 |
| rs56799554 | LINC00910 | 17 | 41456413 | -0.026 | 0.003 | 3.00E-22 | G | A | 0.170 | 94.1 |
| rs75664430 | VAMP2 | 17 | 8064779 | -0.024 | 0.002 | 3.60E-24 | G | C | 0.248 | 102.9 |
| rs144204502 | TK1 | 17 | 76183233 | -0.101 | 0.009 | 3.40E-28 | T | C | 0.013 | 121.2 |
| rs4724 | NAA38 | 17 | 7760397 | -0.055 | 0.003 | 9.80E-69 | A | G | 0.117 | 307.0 |
| rs139669835 | YES1 | 18 | 729871 | -0.061 | 0.011 | 6.10E-09 | T | C | 0.009 | 33.8 |
| rs9955360 | PARD6G | 18 | 78008334 | -0.019 | 0.003 | 2.20E-10 | A | C | 0.869 | 40.3 |
| rs150150565 | ENOSF1 | 18 | 708207 | 0.064 | 0.007 | 6.80E-18 | T | C | 0.021 | 74.3 |
| rs116863223 | ENOSF1 | 18 | 709396 | -0.082 | 0.009 | 2.60E-18 | A | G | 0.012 | 76.2 |
| rs16978028 | LINC01478 | 18 | 42070981 | -0.030 | 0.003 | 8.20E-26 | T | A | 0.144 | 110.3 |
| rs2276182 | POLI | 18 | 51798047 | 0.023 | 0.002 | 2.80E-30 | G | C | 0.403 | 130.7 |
| rs3891167 | TYMS | 18 | 658423 | -0.043 | 0.002 | 1.20E-70 | G | A | 0.253 | 315.8 |
| rs11085072 | SH3GL1 | 19 | 4368142 | -0.013 | 0.002 | 2.60E-08 | T | C | 0.237 | 31.0 |
| rs429358 | APOE | 19 | 45411941 | 0.017 | 0.003 | 3.80E-10 | C | T | 0.154 | 39.2 |
| **SNP** | **Gene** | **Chr** | **Position** | **β** | **SE** | **P-value** | **EA** | **OA** | **EAF** | **F-statistics** |
| rs4530278 | CTD-2540B15.12 | 19 | 33752994 | 0.014 | 0.002 | 1.50E-11 | T | G | 0.598 | 45.5 |
| rs8102497 | MIMT1 | 19 | 57370055 | -0.015 | 0.002 | 1.40E-13 | A | G | 0.432 | 54.7 |
| rs8105767 | AC003973.4 | 19 | 22215441 | 0.033 | 0.002 | 2.50E-50 | G | A | 0.295 | 222.6 |
| rs6054257 | DEFB125 | 20 | 66370 | -0.014 | 0.002 | 1.10E-08 | A | G | 0.794 | 32.7 |
| rs41304832 | ZBTB46 | 20 | 62375508 | 0.061 | 0.009 | 5.00E-11 | A | G | 0.012 | 43.2 |
| rs142426306 | ABHD16B | 20 | 62488152 | -0.050 | 0.005 | 8.70E-21 | T | C | 0.040 | 87.4 |
| rs117512405 | UCKL1 | 20 | 62574274 | -0.079 | 0.008 | 9.50E-22 | A | G | 0.017 | 91.8 |
| rs11699829 | PTK6 | 20 | 62157200 | 0.064 | 0.006 | 1.50E-26 | A | G | 0.034 | 113.7 |
| rs1291143 | SAMHD1 | 20 | 35525640 | 0.049 | 0.003 | 1.80E-69 | C | A | 0.849 | 310.4 |
| rs143190905 | RTEL1 | 20 | 62291767 | -0.072 | 0.004 | 1.60E-85 | T | G | 0.080 | 384.1 |
| rs35640778 | RTEL1 | 20 | 62321128 | -0.209 | 0.007 | 9.59E-195 | A | G | 0.021 | 886.3 |
| rs1003322 | - | 22 | 51072289 | 0.014 | 0.002 | 1.00E-08 | A | C | 0.214 | 32.8 |
| rs6007020 | SMC1B | 22 | 45790132 | 0.014 | 0.002 | 4.80E-12 | C | T | 0.368 | 47.8 |
| rs131797 | - | 22 | 50971631 | 0.024 | 0.002 | 6.80E-25 | T | TAAAAA | 0.236 | 106.2 |
| rs28502153 | GAB4 | 22 | 17469049 | -0.022 | 0.002 | 1.20E-25 | A | C | 0.378 | 109.6 |
| SNP: single nucleotide polymorphism; Chr: chromosome; β: beta coefficient for effect allele; SE: standard error for effect allele; EA: effect allele; OA: other allele; EA: effect allele frequency | | | | | | | | | |  |
|  |  |  |  |  |  |  |  |  |  |  |
