# Supplementary material for: Genetically determined telomere length as a risk factor for hematological malignancies: evidence from Mendelian randomization analysis
Source: Aging (Albany NY). 2024 Mar 6;16(5):4684–98. doi: 10.18632/aging.205625 (PMC10968690; doi:10.18632/aging.205625)
Supplement: Supplementary Table 2 [file aging-16-205625-s003.pdf]

SUPPLEMENTARY TABLES

Supplementary Table 2. Mendelian randomization results of different results.

Outcome	Cases	Controls	MR model	SNP	OR	ll-96%	ul-95%	*P*-value	P(heterogeneity)	P_pleiotropy
Primary lymphoid and hematopoietic malignant neoplasms	7519	299952								
			MR Egger	132	1.787	1.309	2.439	3.713E-04	1.744E-06	
			WM	132	1.842	1.458	2.327	3.042E-07		
			IVW	132	1.517	1.277	1.803		1.326E-06	0.219
			MR-PRESSO	130	1.607	1.358	1.901	3.223E-08	5.669E-05	0.211
Non-follicular lymphoma	2602	299952								
			MR Egger	117	1.796	1.187	2.717	6.512E-03	1.645E-01	
			WM	117	1.793	1.238	2.597	2.019E-03		
			IVW	117	1.585	1.246	2.017	1.736E-04	1.717E-01	0.470
			MR-PRESSO	116	1.677	1.334	2.107	9.234E-06	5.396E-01	0.555
Non-Hodgkin lymphoma	1088	299952								
			MR Egger	132	1.348	0.725	2.504	3.468E-01	5.594E-01	
			WM	132	1.420	0.798	2.526	2.325E-01		
			IVW	132	1.701	1.208	2.396	2.368E-03	5.646E-01	0.378
			MR-PRESSO	132	1.701	1.208	2.396	2.368E-03	5.646E-01	0.378
Follicular lymphoma	1081	299952								
			MR Egger	133	0.831	0.399	1.730	6.213E-01	1.660E-05	
			WM	133	1.395	0.788	2.471	2.535E-01		
			IVW	133	1.012	0.669	1.531	9.566E-01	1.871E-05	0.525
			MR-PRESSO	129	1.280	0.893	1.835	1.792E-01	1.322E-01	0.605
Diffuse large B-cell lymphoma	1010	287137								
			MR Egger	133	1.643	0.865	3.121	1.320E-01	1.318E-01	
			WM	133	1.077	0.631	1.839	7.851E-01		
			IVW	133	1.357	0.944	1.952	9.898E-02	1.380E-01	0.481
			MR-PRESSO	132	1.464	1.035	2.071	3.109E-02	4.109E-01	0.557
Hodgkin lymphoma	780	376497								
			MR Egger	133	1.281	0.642	2.560	4.836E-01	3.973E-01	
			WM	133	1.264	0.674	2.370	4.646E-01		
			IVW	133	1.636	1.106	2.420	1.377E-02	4.041E-01	0.403
			MR-PRESSO	133	1.636	1.106	2.420	1.377E-02	4.041E-01	0.403
Mature T/NK-cell lymphomas	335	299952								
			MR Egger	133	1.574	0.549	4.510	3.998E-01	4.145E-01	
			WM	133	1.568	0.586	4.201	3.705E-01		
			IVW	133	0.694	0.380	1.266	2.332E-01	3.571E-01	0.066
			MR-PRESSO	133	0.694	0.380	1.266	2.332E-01	3.571E-01	0.066
Mantle cell lymphoma	199	287173								
			MR Egger	133	1.651	0.428	6.370	4.684E-01	6.627E-01	
			WM	133	1.734	0.476	6.315	4.035E-01		
			IVW	133	3.134	1.457	6.739	3.452E-03	6.557E-01	0.261

			MR-PRESSO	133	3.134	1.457	6.739	3.452E-03	6.557E-01	0.261
Marginal zone B-cell lymphoma	192	287137								
			MR Egger	133	1.478	0.355	6.146	5.920E-01	2.707E-01	
			WM	133	2.019	0.573	7.111	2.743E-01		
			IVW	133	1.041	0.465	2.328	9.227E-01	2.840E-01	0.559
			MR-PRESSO	133	1.041	0.465	2.328	9.227E-01	2.840E-01	0.559
Lymphoid leukaemia	1493	299952								
			MR Egger	131	3.917	2.126	7.218	2.453E-05	4.347E-01	
			WM	131	3.287	1.970	5.484	5.190E-06		
			IVW	131	2.560	1.865	3.514	5.915E-09	3.976E-01	0.114
			MR-PRESSO	131	2.560	1.865	3.514	5.915E-09	3.976E-01	0.114
Chronic lymphocytic leukaemia	624	287133								
			MR Egger	132	4.734	2.091	10.714	2.846E-04	8.525E-01	
			WM	132	3.456	1.642	7.275	1.089E-03		
			IVW	132	2.797	1.780	4.395	8.208E-06	8.303E-01	0.132
			MR-PRESSO	132	2.797	1.780	4.395	8.208E-06	8.303E-01	0.132
Acute lymphocytic leukaemia	184	287136								
			MR Egger	132	8.887	1.994	39.598	4.856E-03	7.909E-01	
			WM	132	2.214	0.559	8.771	2.578E-01		
			IVW	132	2.655	1.159	6.080	2.092E-02	7.367E-01	0.059
			MR-PRESSO	132	2.655	1.159	6.080	2.092E-02	7.367E-01	0.059
Multiple myeloma	674	376603								
			MR Egger	132	3.470	1.410	8.540	7.694E-03	1.273E-01	
			WM	132	3.053	1.391	6.701	5.406E-03		
			IVW	132	1.852	1.121	3.059	1.616E-02	1.052E-01	0.103
			MR-PRESSO	132	1.852	1.121	3.059	1.616E-02	1.052E-01	0.103

p heterogeneity, *p*-value of Cochrane's *Q*-value in heterogeneity test, P_pleiotropy, *p*-value of MR-Egger intercept.
